# A Novel Wearable Device for Continuous Ambulatory ECG Recording: Proof of Concept and Assessment of Signal Quality

**DOI:** 10.3390/bios9010017

**Published:** 2019-01-21

**Authors:** Christian Steinberg, François Philippon, Marina Sanchez, Pascal Fortier-Poisson, Gilles O’Hara, Franck Molin, Jean-François Sarrazin, Isabelle Nault, Louis Blier, Karine Roy, Benoit Plourde, Jean Champagne

**Affiliations:** 1 Electrophysiology Division, Institut Universitaire de cardiologie et de pneumologie de Québec, Québec, QC G1V 4G5, Canada; Francois.Philippon@fmed.ulaval.ca (F.P.); marina.sanchez.1@ulaval.ca (M.S.); gillesohara@me.com (G.O.); Franck.molin@fmed.ulaval.ca (F.M.); jean-francois.sarrazin@criucpq.ulaval.ca (J.-F.S.); isabellenault@gmail.com (I.N.); Louis.blier@criucpq.ulaval.ca (L.B.); karine.roy@criucpq.ulaval.ca (K.R.); benoit.plourde@criucpq.ulaval.ca (B.P.); Jean.Champagne@fmed.ulaval.ca (J.C.); 2 OMsignal data scientist, Montreal, QC H3T 1J4, Canada; pfortier@omsignal.com

**Keywords:** noninvasive ambulatory rhythm monitoring, Holter, ECG monitoring, telehealth, wearable ECG sensors

## Abstract

Diagnosis of arrhythmic disorders is challenging because of their short-lasting, intermittent character. Conventional technologies of noninvasive ambulatory rhythm monitoring are limited by modest sensitivity. We present a novel form of wearable electrocardiogram (ECG) sensors providing an alternative tool for long-term rhythm monitoring with the potential of increased sensitivity to detect intermittent or subclinical arrhythmia. The objective was to assess the signal quality and R-R coverage of a wearable ECG sensor system compared to a standard 3-lead Holter. In this phase-1 trial, healthy individuals underwent 24-h simultaneous rhythm monitoring using the OMsignal system together with a 3-lead Holter recording. The OMsignal system consists of a garment (bra or shirt) with integrated sensors recording a single-lead ECG and an acquisition module for data storage and processing. Head-to-head signal quality was assessed regarding adequate P-QRS-T distinction and was performed by three electrophysiologists blinded to the recording technology. The accuracy of signal coverage was assessed using Bland-Altman analysis. Fifteen individuals underwent simultaneous 24-h recording. Signal quality and accuracy of the OMgaments was equivalent to Holter-monitoring (84% vs. 93% electrophysiologists rating, *p* = 0.06). Signal coverage of R-R intervals showed a very close overlay between the OMsignal system and Holter signals, mean difference in heart rate of 2 ± 5 bpm. The noise level of OMgarments was comparable to Holter recording. OMgarments provide high signal quality for adequate rhythm analysis, representing a promising novel technology for long-term non-invasive ECG monitoring.

## 1. Introduction

Arrhythmic disorders represent a common referral for cardiology consultations and have a strong impact on resource utilization of health care systems [1]. As many arrhythmias are characterized by intermittent, short-lasting episodes, accurate rhythm documentation is often challenging and a common dilemma for the clinician.

Prolonged ambulatory rhythm monitoring is crucial for the diagnosis of intermittent arrhythmia and the correlation between rhythm and symptoms. Conventional technologies for noninvasive rhythm monitoring include Holter monitoring or external cardiac event recorders. Traditional Holter recording over 24–48 h provides excellent signal quality and high specificity, but is limited by low sensitivity and an overall low diagnostic yield in the vast majority of cases [2]. Despite those obvious limitations, Holter monitoring has been traditionally considered to be the gold standard for ambulatory rhythm monitoring [3]. External cardiac event recorders offer recording durations of 30–90 days and provide improved diagnostic yield compared to Holter monitoring, but are cumbersome and their utility is limited by poor patient compliance [4]. Additional shortcomings include their dependence on appropriate patient-activated event recordings (non-looping external event recorders) and significant wearing discomfort and skin irritation (looping external event recorders).

At present, implantable cardiac monitors (ICMs) represent the only technology for continuous ambulatory long-term monitoring [4]. However, the insertion of ICMs requires minor surgery and is associated with significant costs, and current battery longevities are limited to 3 years. Because of that, novel concepts of wearable ECG sensors have emerged in the past few years. However, most contemporary systems only report heart rates and no ECG tracings and none of it has provided data on the validation of signal quality during direct comparison with Holter monitoring [3,5,6,7,8,9].

Other forms of wearable ECG sensors are already commercially available, but have been typically designed for recreational purposes. At present, clinical experience with those products is limited to pure, numerical detection of the heart rate and respiratory rate [5,6,7,8,9]. Those forms of wearable ECG sensors do not provide ECG rhythm strips and thus, could not be used for distinct rhythm analysis at this point [5,6,7,8,9]. An additional drawback of other contemporary systems is the lack of direct signal validation against Holter recording or another established technology for ambulatory rhythm monitoring. To our knowledge, there is currently no study that has assessed the signal quality of wearable ECG sensors by medical experts (i.e., cardiac electrophysiologists). The utility and potential importance of wearable sensors for rhythm monitoring has been reported previously [10] and the feasibility of cloud-based data transmission/management has been demonstrated by a recent study using a telemedicine surveillance system for atrial fibrillation (AF) screening [11].

The OMsignal system represents a novel, alternative tool for non-invasive ambulatory rhythm monitoring with theoretically unlimited recording capacity and the potential of increased sensitivity to detect intermittent or subclinical arrhythmia. The aim of this study was to validate in a first step the signal quality of the OMsignal garments and signal coverage over 24 h against a standard Holter recording to demonstrate the general utility of the technology for advanced rhythm diagnosis.

## 2. Materials and Methods

### 2.1. Study Population

In this prospective phase-1 trial, healthy individuals underwent 24-h simultaneous rhythm monitoring wearing the OMsignal system together with a 3-lead standard Holter recording (Spiderflash, LivaNova, London, UK) with an ECG sampling rate of 200 samples per second. The study was approved by the local ethics and review board and written informed consent was obtained from all study subjects.

### 2.2. OMgarments–Wearable ECG Sensors

**Figure 1:**
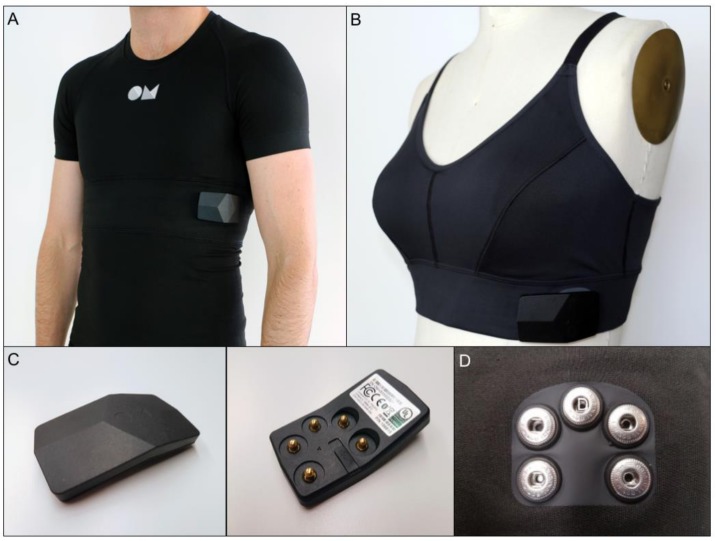
OMgarments are biointeractive wearable ECG sensors. The OMsignal garments represent a novel type of wearable ECG sensors and consist of two different products: The OMshirt (men) (**A**) and the OMbra (women) (**B**). Both garments are intended to be worn as underwear and contain integrated, silicone-based sensors that are in contact with the skin recording a single-lead ECG corresponding to a modified V5-lead. The ECG sensors are connected to the acquisition module (**C**) through five snaps (**D**). The acquisition module has the capacity to save or process ECG signals for real-time or offline analysis.

The OMsignal system (Figure 1) consists of a garment (OMbra for women or OMshirt for men) with integrated sensors and an acquisition module for data recording and processing. The garment contains 3 silicone-based electrodes that are in contact with the skin, recording a single-lead ECG. Two electrodes are situated just below the major pectoral muscles, left and right, providing a modified V5-lead signature. The electrode located at the bottom of the left scapula acts as a right leg lead. A woven wire at the ribcage level records respiratory inductance plethysmography. OMsignal garments must be worn according to a sizing chart to ensure optimal signal quality and wearing comfort (Table S1). The garments used in this project are offered in 4 sizes for both women and men and are machine washable. The ECG sensors are connected to the acquisition module through 5 snaps (Figure 1C–D). The acquisition module has the capacity to save or process ECG signals for offline analysis or real-time monitoring. Data transfer from the acquisition module to a computer simply requires a USB cable. In addition, the acquisition module can also be configured for direct data transfer into a web-based cloud, offering theoretically unlimited recording/storage capacity and direct long-term remote monitoring (Supplementary Figure S1). For this study, the recording time of the acquisition module was set to 24 h with a sampling rate of 125 samples per second.

### 2.3. Validation of Signal Coverage

To validate the overall suitability of the OMsignal system for rhythm analysis and monitoring, we assessed the signal quality and completeness of signal coverage (R-R intervals) over a 24-h recording period. Signal coverage was assessed using automated algorithms and the accuracy compared to the Holter recording was verified using Bland Altman analysis (see below).

R-R intervals were calculated using the built-in algorithms of each device. The OMsignal module embeds a modified Pan-Tompkins real-time QRS detection method with heuristic filtering for misdetections. The comparison of heart rates evaluated the difference between the OMsignal and the Holter recording for every second. This resolution of comparison is crucial to validate the capacity to measure physiological phenomena, such as heart rate variability. In this extent, heart rate was calculated over each R-R and interpolated second by second, which was the smallest time interval allowing accurate comparison. Signal temporal alignment between the Holter and OMsignal consisted of three components: (a) An offset, (b) a constant drift component, and (c) a variable drift component. The offset is to align recordings since they do not start at the same time. It is corrected using the best fit of a cross-correlation of the heart rate between the two signals. The constant time drift is due to hardware clock limitations, where 1 s on one device might correspond to 0.98 or 1.02 s from the other device. The drift between the two signals was calculated through cross-correlation for each hour. The time drift was calculated as the average slope of the 24 offsets. Those changes in time precision are also known to be affected by temperature such that the drift might change if the device is at room vs. body temperature. This phenomenon was taken into consideration by correcting time with the overall drift, but to the hour specific offset. Thus, the nonlinearity was partially compensated by having a specific offset per hour. To ensure maximal accuracy of the planned Bland-Altman analysis, all R-R intervals longer than 2 s without detection of an R-R interval in one device or the other were rejected.

### 2.4. Validation of Signal Quality

Signal quality of the OMgarments was qualitatively assessed regarding the adequate distinction of P-waves, QRS-complexes, T-waves, and noise levels (Appendix A). Among the two ECG leads of the Spiderflash Holter monitoring, the lead with the highest signal quality and lowest rate of high-frequency artefacts was chosen for comparison to the OMshirt/OMbra. Assessment of signal quality was independently performed by 3 experienced electrophysiologists blinded to the recording technology (see Appendix A). For analyses purposes, the ECG signal had its median removed, but no further filter was applied prior to presentation to electrophysiologists.

### 2.5. Statistical Analysis

Qualitative and categorical variables were analyzed using the Fisher exact test or χ^2^-test where appropriate. Continuous variables were tested for normality and are displayed as mean ± standard deviation (SD) or median with interquartile range (IQR) where appropriate. Continuous variables were analyzed using the Student’s *t*-test or Mann-Whitney-U test where appropriate. McNemar’s testing was performed for noninferiority analysis and the comparison of signal quality and noise level assuming a noninferiority margin of 15%. Bland-Altman analysis was used to compare the signal coverage between Holter recordings and the OMsignal system. The Bland-Altman comparison method was selected since both devices were assumed to have inaccuracies to a limited degree at some point of the recording. This method allows the comparison of measurements from two devices without the obligation of one owning the “true” value. The comparison is done by evaluating the relationship between the heart rate differences and the average heart rates of both devices at every second of the recording. The comparison was calculated at the second interval resolution to demonstrate the close beat-to-beat match between the 2 devices to provide the resolution expected from medical ambulatory devices. This metric can only be computed when both devices detected RRs simultaneously, which represented 91% of the recordings’ duration. These values can be summarized in distribution plots and agreement metrics (e.g.,: ±2 bpm agreement when the average heart rate for both devices is 96 bpm). Interobserver agreement between the two blinded electrophysiologists was analyzed performing kappa and R statistics. A *p* value < 0.05 was considered statistically significant. All statistical analysis was conducted using Stata 14.1 Software (StataCorp, College Station, TX, USA).

## 3. Results

### 3.1. Study Population and Wearing Comfort

A total of 15 healthy individuals with a mean age of 41 ± 9 years (60% females) were enrolled and underwent simultaneous 24-h recording. Baseline characteristics of the study population are displayed in Table 1. The overall compliance wearing the two monitoring systems was 100% and there was no premature discontinuation of Holter monitoring or recording using the OMshirt/OMbra. All study subjects indicated that the comfort level of the OMgarments was very high. Study subjects performed usual daily life activities, including exercise and sports. Skin irritations were significantly more common with Holter electrodes and were observed in 7/15 individuals (47%), but only 1/15 individuals (7%) presented skin irritation due to the OMsignal electrode (*p* = 0.03).

**Figure 2:**
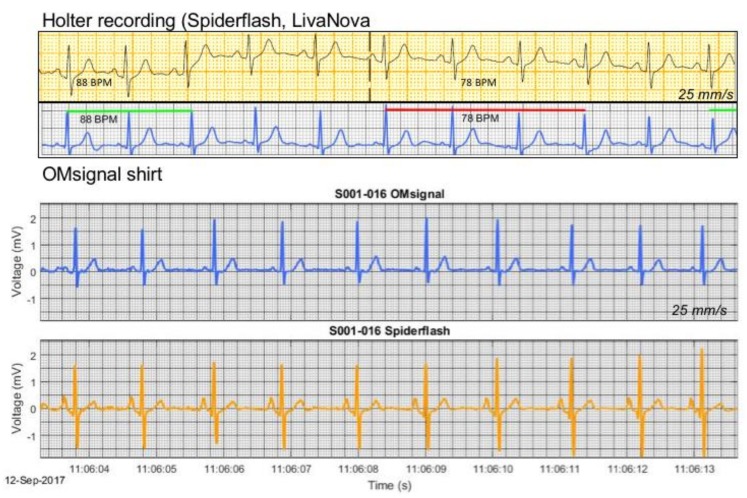
ECG samples of simultaneous recording with OMgarments and Holter. Shown are two samples of simultaneous recording with the OMgarment and a standard 3-lead Holter. The precise beat-to-beat signal coverage of the OMgarment allows even detection of subtle rhythm phenomena, such as respiratory sinus arrhythmia. Note the perfect signal overlay between Holter and garment and high signal quality of the ECG tracings from the OMgarments. The displayed ECG tracings from the OMgarment represent unfiltered signals without additional modification to improve signal quality.

Representative examples of ECG recordings by Holter and the OMsignal system are shown in Figure 2. Even subtle physiologic phenomena, such as respiratory sinus arrhythmia, were readily detectable with this novel recording system.

### 3.2. Assessment of Signal Coverage

**Figure 3:**
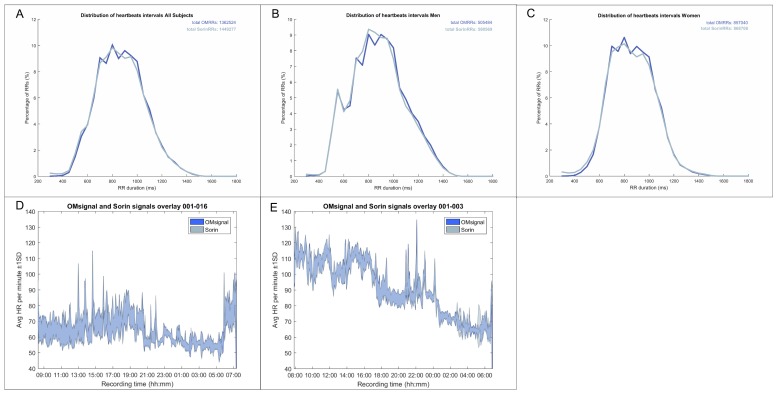
Signal coverage of OMgarments and Holter. Shown is the average signal coverage over 24 h for any given R-R interval (**A**–**C**) and for any given time point during recording (**D**–**E**). Note the almost perfect overlay of signal coverage between OMgarments and Holter recordings.

**Figure 4:**
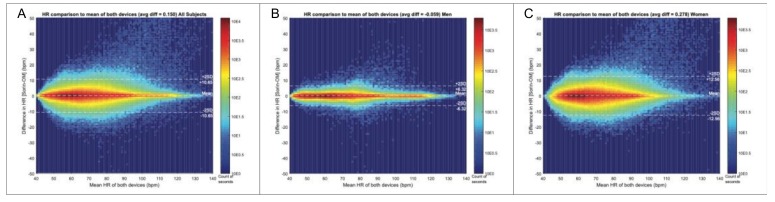
Bland-Altman analysis for signal coverage. (**A**) The overall signal coverage by the OMgarment was excellent, showing an almost perfect overlay compared to standard Holter recording with a mean difference in heart rate of only 2 ± 5 bpm. The quality of signal coverage with OMgarments showed no difference between women (**B**) and men (**C**) and was equivalent to Holter recording.

The distribution of R-R intervals over 24 h is shown in Figure 3 and showed an almost perfect overlay between the OMsignal system and Holter signals over a large range of various R-R intervals (Figure 3A–C) as well as for any given time point over the 24 h recording period (Figure 3D–E). Figure 4 shows Bland-Altman analysis of the signal coverage comparing the heart rate of each second of the recording with OMgarments and Holter. Overall, 90% of the entire recording time of each study subject was suitable for Bland-Altman analysis. The quality of signal coverage resulted in a very close match with the Holter recording, showing a mean difference over 24 h in the heart rate of only 2 ± 5 bpm (Figure 4A). Assuming both devices are not perfect, this level of agreement at the heart beat to heart beat resolution level was unexpected given the challenge of aligning both signals. With an average heart rate of 60 bpm giving an R peaks interval of 1 s, an average difference of 2 ± 5 bpm corresponds to a time difference of 0.034 ± 0.095 s on beat alignment. Bland-Altman analysis did not show any heart rate difference between females and males (Figure 4B–C). Thus, the results showed that R peak detection of the OMshirt/OMbra is equivalent to standard Holter recording.

### 3.3. Assessment of Signal Quality

**Figure 5:**
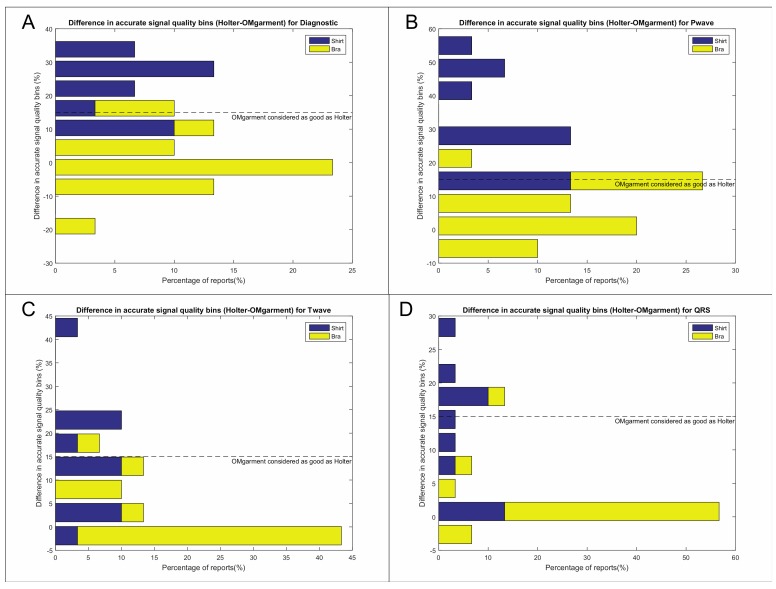
Accuracy of signal morphology by semi-quantitative assessment. Shown are histograms of a Kolmogorov distribution derived from the combined score ranking of the averaged semi-quantitative assessment of the overall morphology accuracy of ECG signals recorded by Holter and the OMgarments. Each histogram displays the proportion and degree of diagnostic bins of recordings with the OMgarments compared to Holter regarding the overall accuracy (**A**) and signal quality of P, QRS, and T (**B**–**D**). Diagnostic bins are presented as stacked columns containing the data for males (OMshirt) and females (OMbra). All diagnostic bins below the prespecified noninferiority margin of 15% indicate that the signal quality of the OMgarment was as good as the Holter recording. (A) The overall signal accuracy was noninferior to the Holter recording, but the results were largely driven by recordings in females. Overall signal quality in males was inferior to Holter recording in up to 75% of recordings, mostly related to high levels of noise (please see Section 3.5) and inferior P-wave quality (**B**). Signal quality of QRS complexes (**C**) and T-waves (**D**) were also significantly better in females compared to males.

Overall signal quality of the OMshirt/-bra was noninferior (Figure 1B) and signal accuracy of Holter-monitoring and OMshirt/-bra was equivalent as per semiquantitative analysis (93% vs. 84%, *p* = 0.06 [Wilcoxon] and McNemar’s chi^2^ = [95% CI 0.69–1.06; *p* = 0.16]). Details of the signal accuracy analysis are displayed in Figure 5 and Table 2. The overall signal accuracy was non-inferior to Holter recording and significantly better in women (OMbra) compared to men (OMshirt) (Figure 5). The signal quality of P-, QRS and T-waves was excellent in women and only 6% of the recordings in women did not meet noninferiority criteria (Table 2, Figure 5). In contrast, noninferior signal quality of QRS complexes and T-waves were only observed in 58% of males (Figure 5C,D) and accurate P-wave morphology in only 25% (Figure 5B). The major reason for the decreased quality of signal morphology in males was related to noise (see below).

### 3.4. Interobserver Agreement

The interobserver agreement regarding the accurate signal quality of P-waves, QRS-complexes, and T-waves was excellent as shown in Table 3. Interobserver agreement was substantial regarding the detection of accurate morphology of P- and T-waves (κ = 0.69 and = 0.63, respectively) and nearly perfect regarding QRS complexes (κ = 1.00).

### 3.5. Noise Level Assessment

Examples of intermittent noise are shown in Figure S3. As rated by the three blinded electrophysiologists, the overall noise levels did not show a significant difference between Holter recordings and recordings with the OMgarments (Figure S4A). The median recording percentage displaying no or only minimal intermittent, short-lasting noise was 81% for all Holter recordings and 69% for recordings with the OMgarment (*p* = 0.26). Thus, the vast majority of the 24 h of recordings was rated as low to very low noise levels on both devices. Noise levels during Holter recordings were similar between males and females (*p* = 0.41) (Figure S4C). However, recordings with the OMgarments showed higher noise levels in males compared to females (*p* = 0.01) (Figure S4B). The median percentage of recordings without or minimal intermittent noise was 78% in females (OMbra) compared to 53% in males (OMshirt). Physical activities, including sports participation, did not result in an increase of the noise level or affect the signal quality. The main difference in signal quality between OMgarments and Holter recordings was related to the inferior quality of P-wave morphology in males.

## 4. Discussion

Extended ambulatory rhythm monitoring is a key element for the diagnosis and management of various rhythm disorders [4,12]. However, the diagnostic yield of ambulatory rhythm monitoring highly depends on the frequency of symptoms and the recording duration [13,14]. At present, long-term ambulatory rhythm monitoring is limited to external cardiac event recorders (up to 90 days) and implantable cardiac monitors (up to 3 years) [12,13,14].

The OMgarments represent a novel form of wearable ECG sensors providing equivalent signal quality and may be an alternative tool for ambulatory rhythm monitoring. The garments include bras for women and shirts for men that contain integrated electrodes detecting a real-time single-lead surface ECG. In the present study, we demonstrated that the OMgarments provide excellent signal coverage equivalent to standard Holter recording combined with a high signal quality. A particular strength of this phase-1 validation study was the direct, real-time head-to-head recording with OMgarments and a standard 3-lead Holter in each study subject–an approach that has been rarely performed in previous studies introducing novel non-invasive monitoring devices. Using this synchronized recording approach, we showed a very close overlay of R-R coverage (within 2 bpm over 24 h) between the OMgarments and the standard Holter. To the best of our knowledge, no previous study comparing non-invasive monitoring devices has ever reported such a close signal coverage. Excellent R-R coverage is a crucial element for reliable rhythm diagnosis and our study has shown that the OMgarments do not miss heart beats that might have otherwise been recorded by looping event recorders or standard Holter monitoring.

The garments also provided high signal quality that was non-inferior compared to Holter recording. Accurate distinction of P-waves, QRS-complexes, and T-waves was present in most study subjects and based on a semi-quantitative evaluation by experienced electrophysiologists. The overall better signal quality in women is most likely related to a better skin contact of the bra electrode compared to the shirt electrode in men. Although the garment size was selected based on individual under-chest circumference and bust circumference, the overall fit was better in women. Those issues are currently being addressed in the design of the next generation of OMshirts. Changes of the electrode position and size as well as improved noise filter algorithms should overcome the observed differences in noise levels between bras and shirts in the future.

An additional advantage of the garment in this study is the high wearing comfort and minimal risk of skin irritation (7% in this study) compared to conventional electrodes of Holter/looping external event recorders (47% in this study) or adhesive cardiac monitoring patches (personal clinical observations). Moreover, conventional looping external event recorders are cumbersome and interfere with showering, sports, and other daily life activities, resulting in poor patient compliance and reduced diagnostic yield [4]. Adhesive cardiac monitor patches are characterized by ease-of-use, increased wearing comfort, and allow showering [15], but have limited recording durations (7–30 days) compared to conventional external loop recorders or implantable cardiac monitors [4,16,17] This study was intentionally limited to a recording period of 24 h, but the built-in capacity of signal transmission from the garment into a web based cloud provides already the technical basis for long-term monitoring, which will be studied in an upcoming multicenter trial. Also, the signal quality of the garments remains stable over prolonged recording periods, including washing (unpublished results), whereas there are some concerns about decreased signal quality and loss of automated rhythm analysis in adhesive monitor patches [18].

At present, implantable cardiac monitors (ICMs) represent the only device of ambulatory long-term monitoring beyond a period of 3 months. The utility and diagnostic accuracy of ICMs is well-established and the recent model of one manufacturer has been miniaturized to a volume of only 1.2 cm^3^ [4,19]. However, ICMs are invasive with a small, but real risk of complications and the recording capacity is limited to 57 min, whereas the wearable ECG sensors, like the OMgarments, provide potentially unlimited recording (Steinberg et al., 2017). Moreover, the insertion of an ICM is associated with significant upfront costs compared to wearable ECG sensors, like the garments of this study.

The potential spectrum of clinical applications for wearable ECG sensors, like the OMgarments, is broad, including diagnosis of unexplained syncope or palpitations, screening for subclinical atrial fibrillation, monitoring of heart rate variability and antiarrhythmic treatment effects, or screening for recurrent arrhythmia after ablation procedures. From a population health perspective, the screening for subclinical atrial fibrillation (AF) has gained wide interest, since two recent large-scale prospective studies have demonstrated that occult AF is a major source of cryptogenic ischemic stroke [20,21]. In the pivotal CRYSTAL-AF and EMBRACE-AF study, subclinical AF was detected in 12–16% of study subjects over 3–12 months using an ICM (CRYSTAL-AF) or external non-looping event recorder (EMBRACE-AF) [20,21]. Extended monitoring up to 3 years through an ICM in the CRYSTAL-AF trial was even associated with an increase of subclinical AF up to 30% of all study subjects. Based on those results, long-term rhythm monitoring to search for AF has been recommended in all individuals with cryptogenic stroke [22], making wearable ECG sensors, like the OMgarments, an attractive, easy-to-use, and less costly alternative. In a similar manner, wearable ECG sensors may enrich the arsenal of ambulatory long-term monitoring tools to assess medical and interventional treatment effects in various arrhythmia as well as risk prediction in structural heart disease or inherited arrhythmia [4,23,24].

One of the main challenges of wearable ECG sensors is the use of dry contact electrodes on a recording surface subject to movement artifacts. The current study demonstrates that OMsignal garments have a similar performance to a Holter monitor. As previously shown, such garments may have great potential in clinical settings. The cost of an OMsignal unit is quite low (180$ US), allowing a wider distribution of ambulatory monitoring. Patient set up and follow up are also simplified. Electrode placement is standardized using a sizing chart and once a patient is properly equipped, garments provide recordings of reproducible signal quality. The follow up is simplified as wireless transmission allows remote data transfer to a secured cloud and faster data processing. Combined with the rechargeable module, it would allow prolonged monitoring periods although this has yet to be demonstrated. Another challenge of prolonged recordings is the interaction between skin and the dry contact electrodes, which is minimized by the integrated electrodes of the garment. 

Wearable ECG sensors, like the OMgarments, are designed to integrate medical data acquisition into usual daily life activities. The emergence of artificial intelligence to monitor cardiac conditions opens the door for novel strategies of prolonged ambulatory monitoring [25,26]. Biointeractive garments, such as the presented one, could leverage these novel technologies.

### Limitations

This is a single-center study for a proof-of-concept that was conducted in healthy individuals. The sample size of this study is very modest, however, the crossover design with simultaneous Holter recording assured a true head-to-head design transforming each study subject into its own control. Performing a beat-by-beat analysis for signal coverage resulted in a large amount of quantitative data for the overall analysis. Therefore, we do not believe that the small sample size may have influenced the overall message of this study. All study subjects had a normal body mass index and there may be some uncertainty about the recording accuracy in individuals with extreme body weights. Overall signal quality was better in women, which is related to the better wearing fit of the OMbra. Despite that difference, the overall signal quality in men was still sufficient to establish a rhythm diagnosis in the vast majority of cases and there was no difference between males and females with regards to the accurate detection of heart rate and heart rate variability over 24 h. The present study was limited to a recording period of 24-h and a consistent signal quality over longer monitoring periods has yet to be demonstrated. An upcoming multicenter trial will address the utility of the wearable ECG sensors for mid- and long-term monitoring.

## 5. Conclusions

In summary, the OMgarments (OMshirt and OMbra) represent a novel class of wearable ECG sensors characterized by its ease to use and a built-in capacity of short-term and long-term rhythm monitoring. Signal accuracy of the OMgarments is equivalent to standard Holter recording. The OMgarments provide high signal quality for adequate rhythm analysis and represent a promising technology for widespread use in ambulatory non-invasive ECG monitoring.

## Figures and Tables

**Table 1:** Baseline characteristics of the study population.

	All (N = 15)
Age, years	41 ± 9
Females, n (%)	9 (60)
Height, cm	168 ± 8
Weight, kg	70 ± 9
BMI, kg/m^2^	24.8 ± 2
Under-chest circumference, cm	87 ± 8
Bust circumference, cm	95 ± 7
Resting heart rate, bpm	65 ± 14
Systolic blood pressure, mmHg	123 ± 12
Diastolic blood pressure, mmHg	75 ± 5

BMI = body mass index

**Table 2:** Proportion of noninferiority of ECG signal quality of OMgarments.

Percentage of Non-Inferiority Compared to Holter Recording	Males (N = 6)	Females (N = 9)
P-waves	17%	94%
QRS complexes	58%	94%
T-waves	58%	94%

**Table 3:** Interobserver agreement for accurate ECG morphology of OMgarments.

ECG Parameter	Kappa (Unweighted)	Kappa (Quadratic Weighting)	Observed Proportions of Agreement	95% CI
P-wave	0.69	0.87	0.87	(0.58–0.98)
QRS complex	1.00	1.00	1.00	(0.75–1.00)
T-wave	0.63	0.63	0.93	(0.66–1.00)

## Supplementary Materials

The following are available online at http://www.mdpi.com/2079-6374/9/1/17/s1, Figure S1: Cloud-based data transmission; Figure S2: Smart phone application; Figure S3: Noise samples; Figure S4: Noise level comparision; Table S1. Sizing chart of OMgarments.

## Appendix A

### Supplementary Methods

#### OMgarments–Wearable ECG Sensors

OMgarments (OMshirt and OMbra) are wearable ECG sensors and are intended to be used as underwear, as the ECG sensor must be in direct contact with the skin. All OMgarments are machine washable and are designed for long-term use (up to several years). The OMgarments must be worn according to a sizing chart based on the under-chest circumference. At present, four different sizes of OMgarments are available (Table S1). Adequate fitting of the garment is crucial to obtain accurate signal quality.

#### Data Recording and Transmission

The placement of the electrodes results in a single-lead ECG similar to V5. The analog ECG signals are recorded by the acquisition module (Figure 1C) and converted into digital signals. The acquisition module can either process and send data or save data in the module’s internal memory. The components of the acquisition module comprise a signal converter (analog to digital), a 3 axes accelerometer, a battery, a memory unit, a Bluetooth antenna, and a microprocessor. The module interfaces with the garment through five stainless steel snaps (or press studs) common in clothing fastening (Figure 1D), thus achieving high stability and an extremely low risk of inadvertent detachment. The battery of the acquisition module can last up to 30 h in raw recording configuration. A USB connector located at the module serves to access the memory and to charge the battery. The module design prevents the simultaneous connection of the module with both the garment and USB cable. Once connected through the USB to a computer, raw ECG and respiration signals saved on the module can be downloaded for analyses. Data will remain on the module until another recording session is initiated. In addition to internal data storage, the acquisition module can also be configured for direct data transfer into a web-based cloud, offering theoretically unlimited recording/storage capacity as well as offline and real-time rhythm analysis (Figure S1). Data acquisition is controlled wirelessly through Bluetooth commands using a proprietary communication protocol and can be performed with a smart phone. A smart phone application is used to select the module and start rhythm recording (Figure S2). The sampling rate of ECG is usually at 250 samples per second. The R-Rs events are detected at the module level through a modified Pan-Tompkins algorithm [27]. For this study, the acquisition rate was set to 125 samples per second to lighten data management through USB. The interpolated ECG signal was introduced through the RR peak detection algorithm in a module simulator on the computer.

#### Assessment of Signal Quality

Signal quality and accuracy of distinct recognition of P-waves, QRS-complexes, and T-waves was performed by three independent, experienced electrophysiologists who analyzed all Holter and OMsystem tracings and were blinded to the underlying recording technology. The 24 h of each Holter and garment recording were divided into 96 blocks of 15 min recording time. Each recording block was assessed for noise level and overall accuracy to establish a diagnosis of the underlying rhythm. In addition, rhythm strips of the last 7 s of each hour were extracted for semiquantitative analysis of accurate morphology of P-waves, QRS complexes, and T-waves. The quality of distinct P-, QRS-, and T-wave morphology was classified into three categories for each parameter: (1) Dominant = excellent signal quality, easy rhythm diagnosis; 75–100% of all beats of the rhythm strip exhibiting distinct and well defined P, QRS and T; (2) significant = acceptable signal quality, rhythm diagnosis can be established for 50–75% of the tracing; and (3) inadequate = accurate ECG morphology present in less than 50% of the tracing-signal quality not useful for rhythm diagnosis. Both devices were compared for each subject, providing one entry per electrophysiologist. The distribution of device difference was expressed in a distribution histogram, with negative values demonstrating more observations of features on the OMsignal system. The percentage and degree of morphology bins deviating from Holter morphology was calculated using a noninferiority margin of 15%, which was based on clinical judgement in the absence of any historical or comparative data in the literature.

#### Noise Level Assessment

Noise was defined as the recording of any isolated or sustained, nonphysiological high-frequency signals with abnormally low or high amplitudes causing baseline deviations or P-QRS-T masking that did not correspond to a normal electrophysiological signal generated by the heart. Noise levels were also assessed by three experienced electrophysiologists who were blinded to the corresponding recording technology. To avoid any contamination of the morphology analysis, we also included clipped or truncated signal amplitudes that may have corresponded to extremely high-volted QRS complexes. Fluctuations or shifts of the isoelectric baseline of ≥50% of the tracing were also coded as noise. Given those severe criteria, minimal noise was defined as a tracing without any noise or <25% noise recordings of the rhythm strip. Since the OMgarments are a tool for long-term monitoring, a minimal noise level of <25% was felt to be acceptable given the large amount of data/heart beats that would still be available in a worst-case scenario of up to 25% uninterpretable beats.

## References

[1] Tang D.H., Gilligan A.M., Romero K. (2014). Economic burden and disparities in healthcare resource use among adult patients with cardiac arrhythmia. Appl. Health Econ. Health Policy.

[2] Steinberg C., Bennett M.T., Krahn A.D., Kowey P., Piccini J.P., Naccarelli G. (2017). Extended ECG Monitoring. Cardiac Arrhythmias, Pacing and Sudden Death.

[3] (1997). Survivors of out-of-hospital cardiac arrest with apparently normal heart. Need for definition and standardized clinical evaluation. Consensus Statement of the Joint Steering Committees of the Unexplained Cardiac Arrest Registry of Europe and of the Idiopathic Ventricular Fibrillation Registry of the United States. Circulation.

[4] Tomson T.T., Passman R. (2017). Current and Emerging Uses of Insertable Cardiac Monitors: Evaluation of Syncope and Monitoring for Atrial Fibrillation. Cardiol. Rev..

[5] Cheng Y., Ye Y., Hou M., He W., Li Y., Deng X. (2018). A Fast and Robust Non-Sparse Signal Recovery Algorithm for Wearable ECG Telemonitoring Using ADMM-Based Block Sparse Bayesian Learning. Sensors.

[6] Elliot C.A., Hamlin M.J., Lizamore C.A. (2017). Validity and reliability of the Hexoskin(R) wearable biometric vest during maximal aerobic power testing in elite cyclists. J. Strength Cond. Res..

[7] Lin W.Y., Ke H.L., Chou W.C., Chang P.C., Tsai T.H., Lee M.Y. (2018). Realization and Technology Acceptance Test of a Wearable Cardiac Health Monitoring and Early Warning System with Multi-Channel MCGs and ECG. Sensors.

[8] Lu K., Yang L., Seoane F., Abtahi F., Forsman M., Lindecrantz K. (2018). Fusion of Heart Rate, Respiration and Motion Measurements from a Wearable Sensor System to Enhance Energy Expenditure Estimation. Sensors.

[9] Villar R., Beltrame T., Hughson R.L. (2015). Validation of the Hexoskin wearable vest during lying, sitting, standing, and walking activities. Appl. Physiol. Nutr. Metab..

[10] Majumder S., Mondal T., Deen M.J. (2017). Wearable Sensors for Remote Health Monitoring. Sensors.

[11] Chen Y.H., Hung C.S., Huang C.C., Hung Y.C., Hwang J.J., Ho Y.L. (2017). Atrial Fibrillation Screening in Nonmetropolitan Areas Using a Telehealth Surveillance System With an Embedded Cloud-Computing Algorithm: Prospective Pilot Study. JMIR Mhealth Uhealth.

[12] Steinberg J.S., Varma N., Cygankiewicz I., Aziz P., Balsam P., Baranchuk A., Cantillon D.J., Dilaveris P., Dubner S.J., El-Sherif N. (2017). 2017 ISHNE-HRS expert consensus statement on ambulatory ECG and external cardiac monitoring/telemetry. Heart Rhythm.

[13] Krahn A.D., Andrade J.G., Deyell M.W. (2013). Selecting appropriate diagnostic tools for evaluating the patient with syncope/collapse. Prog. Cardiovasc. Dis..

[14] Zimetbaum P., Goldman A. (2010). Ambulatory arrhythmia monitoring: Choosing the right device. Circulation.

[15] Smith W.M., Riddell F., Madon M., Gleva M.J. (2017). Comparison of diagnostic value using a small, single channel, P-wave centric sternal ECG monitoring patch with a standard 3-lead Holter system over 24 hours. Am. Heart J..

[16] Barrett P.M., Komatireddy R., Haaser S., Topol S., Sheard J., Encinas J., Fought A.J., Topol E.J. (2014). Comparison of 24-hour Holter monitoring with 14-day novel adhesive patch electrocardiographic monitoring. Am. J. Med..

[17] Tung C.E., Su D., Turakhia M.P., Lansberg M.G. (2014). Diagnostic Yield of Extended Cardiac Patch Monitoring in Patients with Stroke or TIA. Front. Neurol..

[18] Cheung C.C., Kerr C.R., Krahn A.D. (2014). Comparing 14-day adhesive patch with 24-h Holter monitoring. Future Cardiol..

[19] Purerfellner H., Sanders P., Pokushalov E., Di Bacco M., Bergemann T., Dekker L.R., Reveal L.U.S.I. (2015). Miniaturized Reveal LINQ insertable cardiac monitoring system: First-in-human experience. Heart Rhythm.

[20] Gladstone D.J., Sharma M., Spence J.D., Committee E.S., Investigators (2014). Cryptogenic Stroke and Atrial Fibrillation. N. Engl. J. Med..

[21] Sanna T., Diener H.C., Passman R.S., Di Lazzaro V., Bernstein R.A., Morillo C.A., Rymer M.M., Thijs V., Rogers T., Beckers F. (2014). Cryptogenic stroke and underlying atrial fibrillation. N. Engl. J. Med..

[22] Widimsky P., Doehner W., Diener H.C., Van Gelder I.C., Halliday A., Mazighi M. (2018). The role of cardiologists in stroke prevention and treatment: Position paper of the European Society of Cardiology Council on Stroke. Eur. Heart J..

[23] Malmo V., Nes B.M., Amundsen B.H., Tjonna A.E., Stoylen A., Rossvoll O., Wisloff U., Loennechen J.P. (2016). Aerobic Interval Training Reduces the Burden of Atrial Fibrillation in the Short Term: A Randomized Trial. Circulation.

[24] Exner D.V., Kavanagh K.M., Slawnych M.P., Mitchell L.B., Ramadan D., Aggarwal S.G., Noullett C., Van Schaik A., Mitchell R.T., Shibata M.A. (2007). Noninvasive risk assessment early after a myocardial infarction the REFINE study. J. Am. Coll. Cardiol..

[25] Clifford G.D., Liu C., Moody B., Lehman L.H., Silva I., Li Q., Johnson A.E., Mark R.G. (2017). AF Classification from a Short Single Lead ECG Recording: The PhysioNet/Computing in Cardiology Challenge 2017. Comput. Cardiol..

[26] Acharya U.R., Fujita H., Oh S.L., Hagiwara Y., Tan J.H., Adam M. (2017). Application of deep convolutional neural network for automated detection of myocardial infarction using ECG signals. Inf. Sci..

[27] Pan J., Tompkins W.J. (1985). A real-time QRS detection algorithm. IEEE Trans. Biomed. Eng..

