# Supplementary material for: A Novel Wearable Device for Continuous Ambulatory ECG Recording: Proof of Concept and Assessment of Signal Quality
Source: Biosensors (Basel). 2019 Jan 21;9(1):17. doi: 10.3390/bios9010017 (PMC6468449; doi:10.3390/bios9010017)
Supplement: Supplementary file 1 [file biosensors-09-00017-s001.pdf]

Table S1. Sizing chart of OMgarments.

	Under-chest circumference (cm)	
**Size**	**Men**	**Women**
XS	90–96	66–71
S	96–101	71–78
M	101–106	78–85
L	106–112	85–96

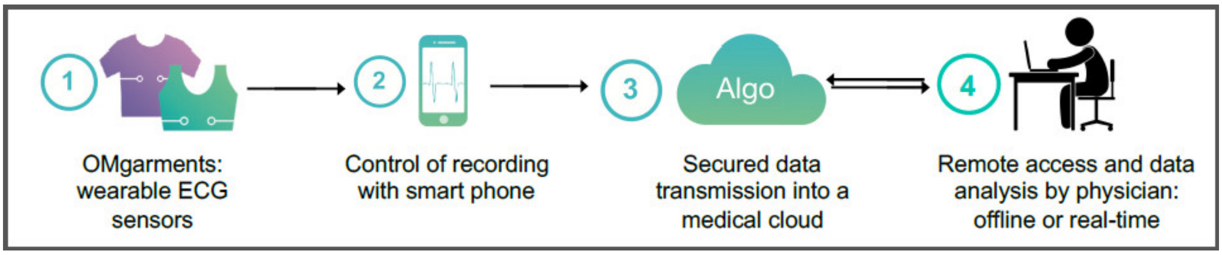

Figure S1. Cloud-based data transmission and remote rhythm analysis.

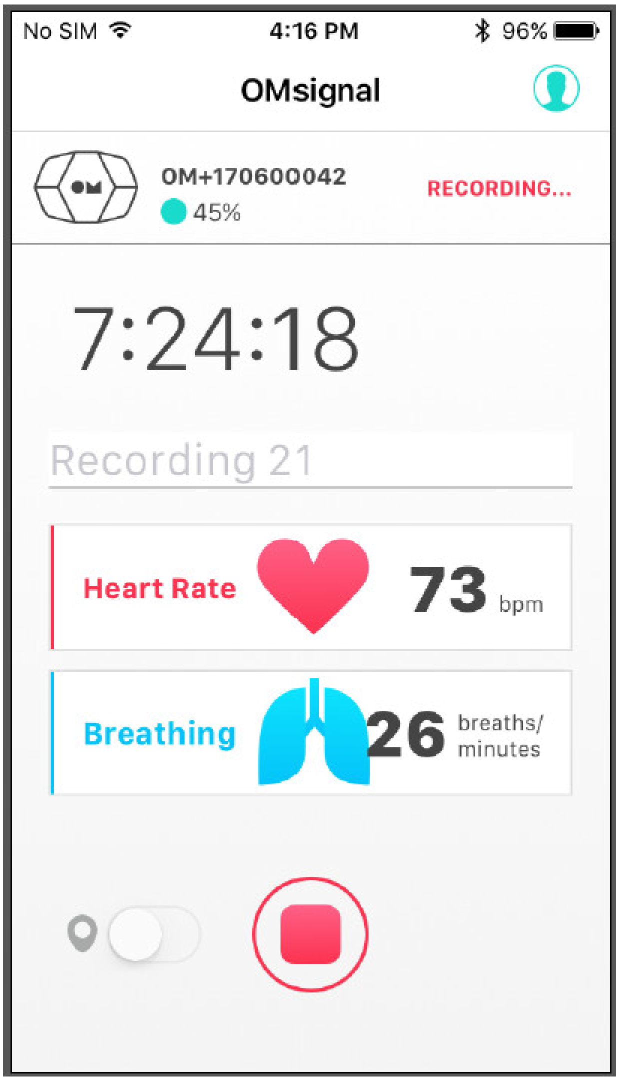

Figure S2. Smartphone application for real-time monitoring and data transmission.

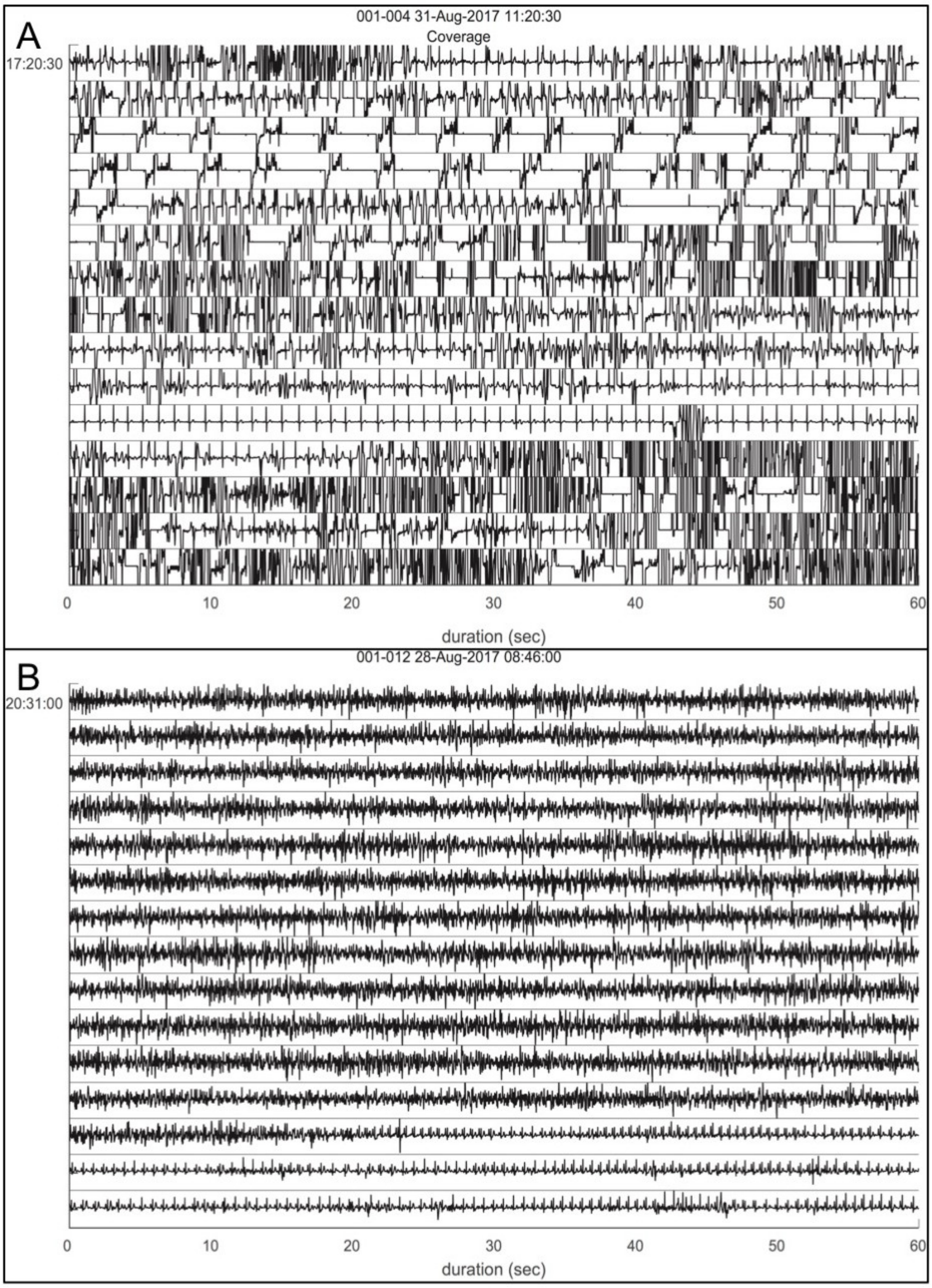

Figure S3. Examples of noise recordings. (A) Shown is a representative example of noise recording with the OMgarment in a male study subject. Noise detection was caused by suboptimal electrode – skin contact. (B) Example of noise recording from a Holter monitoring (Spiderflash, LivaNova). Noise recording was related to moving artefacts.

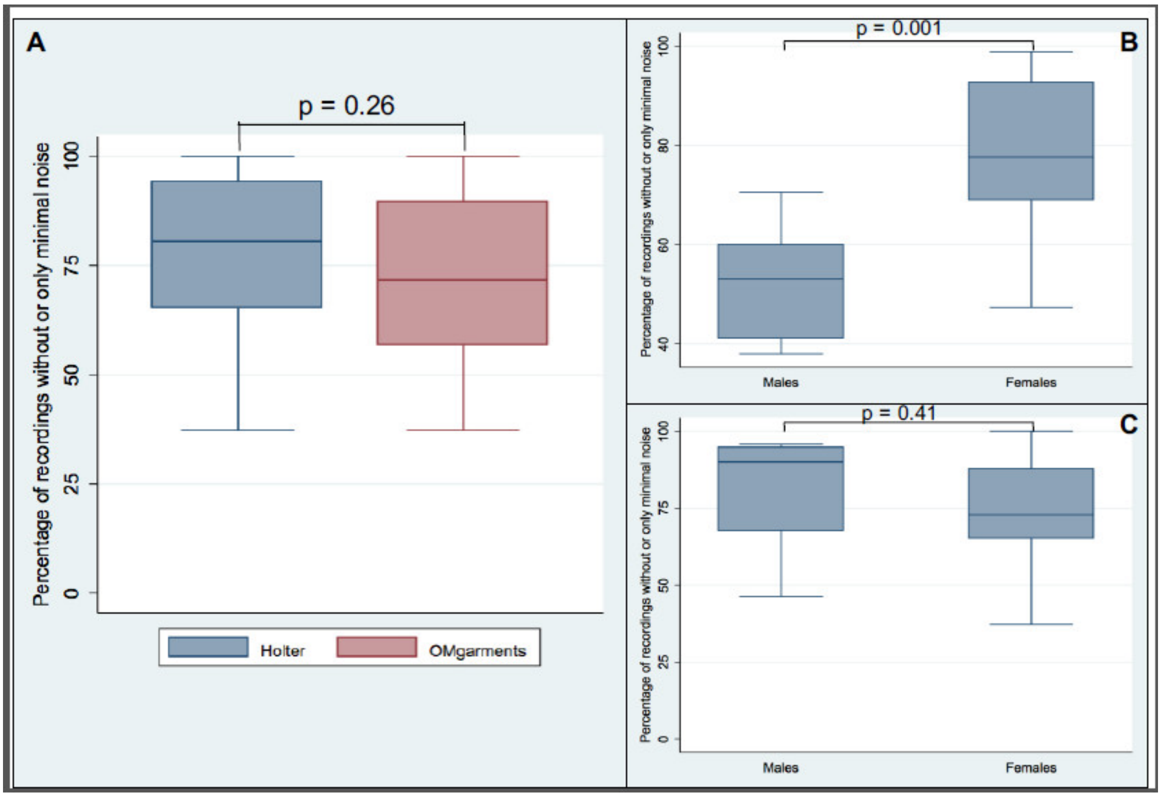

Figure 4. Comparison of noise levels between OMgarments and Holter. Shown are box plots of the overall percentages of ECG recordings without noise or only minimal noise. Minimal noise was defined as < 25% of noise recording over each analysis block of 15 minutes for a 24-h recording period. (A) The overall noise level did not show any difference between OMgarments and Holter recording. (B-C) Among the OMgarments, significantly more noise was recorded in males compared to females (B), whereas Holter recordings did not show any sex-related differences (C).
